# CFVisual: an interactive desktop platform for drawing gene structure and protein architecture

**DOI:** 10.1186/s12859-022-04707-w

**Published:** 2022-05-13

**Authors:** Huilong Chen, Xiaoming Song, Qian Shang, Shuyan Feng, Weina Ge

**Affiliations:** 1 School of Life Science, North China University of Science and Technology, Tangshan, 063210 Hebei China; 2 School of Information Science and Technology, Yanching Institute of Technology, Langfang, 065000 Hebei China

**Keywords:** CFVisual, Gene structure, Motif, Domain, Promoter

## Abstract

**Background:**

When researchers perform gene family analysis, they often analyze the structural characteristics of the gene, such as the distribution of introns and exons. At the same time, characteristic structural analysis of amino acid sequence is also essential, for example, motif and domain features. Researchers often integrate these analyses into one image to dig out more information, but the tools responsible for this integration are lacking.

**Results:**

Here, we developed a tool (CFVisual) for drawing gene structure and protein architecture. CFVisual can draw the phylogenetic tree, gene structure, and protein architecture in one picture, and has rich interactive capabilities, which can meet the work needs of researchers. Furthermore, it also supports arbitrary stitching of the above analysis images. It has become a useful helper in gene family analysis. The CFVisual package was implemented in Python and is freely available from https://github.com/ChenHuilong1223/CFVisual/.

**Conclusion:**

CFVisual has been used by some researchers and cited by some articles. In the future, CFVisual will continue to serve as a good helper for researchers in the study of gene structure and protein architecture.

## Background

With the continuous sequencing of more and more genomes of plants and animals, a large number of genome annotation files have been produced, which are generally in formats such as GFF3 and GTF. Researchers often need to obtain information about gene structure of some gene sets (such as gene families) from these annotation files and display these exon–intron structure graphically. This can help researchers to understand the composition and position of gene exons and introns, and help to advance the understanding of gene variable splicing. Moreover, in conjunction with phylogenetic analysis, it also helps to understand gene evolution. At present, the better drawing tool is GSDS [1]. Unfortunately, it does not fully satisfy the requirements of researchers for graphics. The defects are as follows: the phylogenetic tree cannot be classified and colored, specific numerical information cannot be provided, and the website is often inaccessible, etc.

Motifs and domains are the functional units and characteristic structures of amino acid sequences, and are often identified by tools such as MEME and Pfam/NCBI-CDD/SMART [2–5]. Displaying these motifs and domains along a line helps folk understand the structure of the protein sequence. Comparing with other protein sequences is helpful to find out the conserved parts and difference sites. Moreover, combined with the phylogenetic tree, it is helpful to study the evolution of motifs and domains. When conducting gene family analysis, researchers often need to splice the gene structure map with the motif and/or domain location distribution map into one map for display, so as to obtain more information. Therefore, researchers need to use Adobe Illustrator, Adobe Photoshop or other image editing software to stitch the images. To the best of our knowledge, this work is time-consuming and tedious. Therefore, it is important to develop a suitable tool to avoid this situation.

## Methods

We used the Python language to write the software implementation logic, then used the Python language PySide2 library to implement the software GUI interface, and then used the Python language matplotlib library to visualize the data via our own logic. Finally, We used the Pyinstaller library in the Python language to complete the creation of the CFVisual platform.

In order to better reflect the advantages of CFVisual, we downloaded the latest rice genome data from the rice database (http://rice.uga.edu/) [6], including the whole genome protein sequence and GFF3 annotation file, and then used HMMER software (parameter threshold was set to 1e-10) based on the pectinesterase domain Hidden Markov model (PF01095.19) to identify the candidate sequences of rice PME protein [7]. Finally, all candidate protein sequences were determined by Pfam (https://pfam.xfam.org/), NCBI-CDD (https://www.ncbi.nlm.nih.gov/cdd), and SMART (http://smart.embl-heidelberg.de/) databases, and only protein sequences that contain the pectinesterase domain are considered members of the PME gene family.

After that, we wrote a Python script (https://github.com/ChenHuilong1223/CFVisual/) to extract the amino acid sequences and GFF3 annotation information of rice PMEs. The amino acid sequences of rice PME were analyzed by MEGA X [8], MEME (https://meme-suite.org/meme/), Pfam, NCBI-CDD, and SMART tools to generate the result file. Finally, these results were visualized using CFVisual.

## Results

### Function overview, usage, and illustrative examples

In the functional aspect, CFVisual can be divided into three parts, namely gene structure level, protein architecture level, and classification and coloring of phylogenetic tree.

### Gene structure

**Fig. 1:**
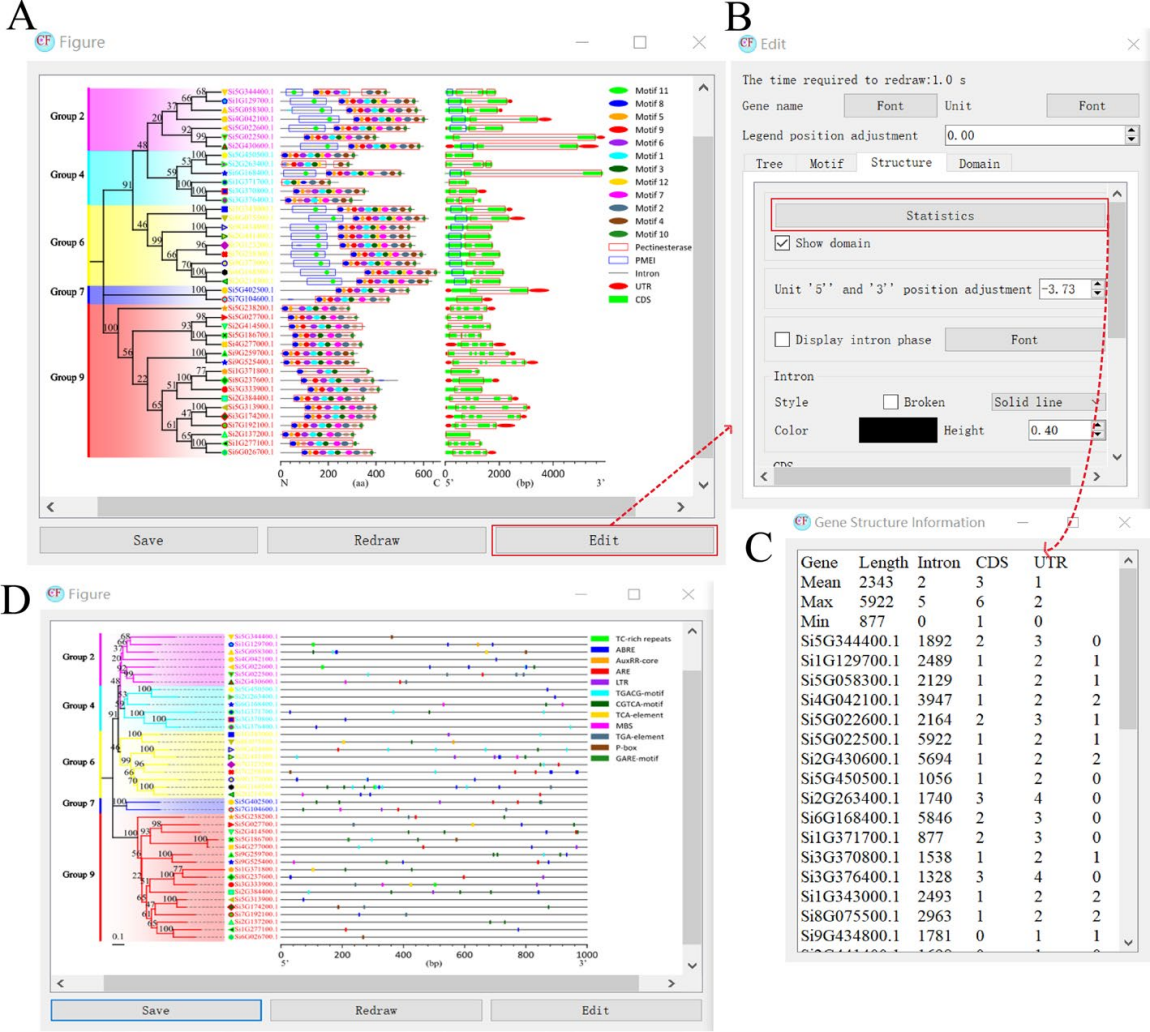
Drawing function and core interface of CFVisual. **a** Classic stitching diagram in structural analysis (tree + motif + gene structure + domain diagram). **b** User interaction window. Each tab corresponds to the control interface of a graphical part. **c** The basic statistical details on structural elements of genes. **d** The subgraph of promoter

Users can provide GFF3, GTF or BED files, and then use CFVisual to draw the picture. In the interface shown in Fig. 1b, users can set the style of each feature, such as color, shape, thickness, etc. Clicking the “Statistics” button to make CFVisual automatically count the length of gene, the number of introns, utrs, cds, and other quantitative information (Fig. 1c). Of course, users can also add other information, including domains and signal peptides, etc. (Fig. 1a). Using the combined form of rectangular boxes helps researchers intuitively judge which cds fragments encode the domain and the presence of introns.

Regarding the promoter map (Fig. 1d), users provide location results from PlantCare [9] and other tools for predicting the position of *cis*-acting elements and CFVisual will read out all *cis*-acting elements at once, which can be selectively displayed according to needs.

### Protein architecture

The preparation file for drawing the motif diagram (Fig. 1a) is the result file predicted by the MEME tool. Compared with some conventional motif visualization tools, the advantages of CFVisual are as follows. First of all, the software completely reproduces the results of MEME and realizes that the height of the rectangular box representing the motif is negatively correlated with the *p* value. The lower the height, the higher the *p* value, and the lower the credibility of the predicted motif. Secondly, the result of “Scanned Sites” can be displayed in the form of transparent rectangular boxes. At last, users can selectively display motif units that need to be studied.

The preparation file of the domain map is the result file of NCBI-CDD, Pfam or SMART. Users can still selectively display the domains that need to be studied. Another advantage of CFVisual is that the structure domain can be superimposed on the motif diagram in the form of a rectangular box (Fig. 1a), so that researchers can intuitively judge the location distribution relationship of motifs and domains.

### Classification and coloring of phylogenetic tree

While studying gene structure and protein architecture, researchers often joint a phylogenetic tree to study the evolution of structures. Here, CFVisual supports this demand well. Users only needs to provide the tree file in Newick format to be recognized by CFVisual and can draw the picture easily (Fig. 1a). After that, researchers can use the “Tree Edit Tab” to classify and color the phylogenetic tree, and finally produce high-definition bitmaps and/or editable vector graphics that meet publication quality.

### Illustrative examples

To better reflect the above advantages of CFVisual, we take the gene structure, motif, and domain drawing results of the PME gene family of rice as an example.

**Fig. 2:**
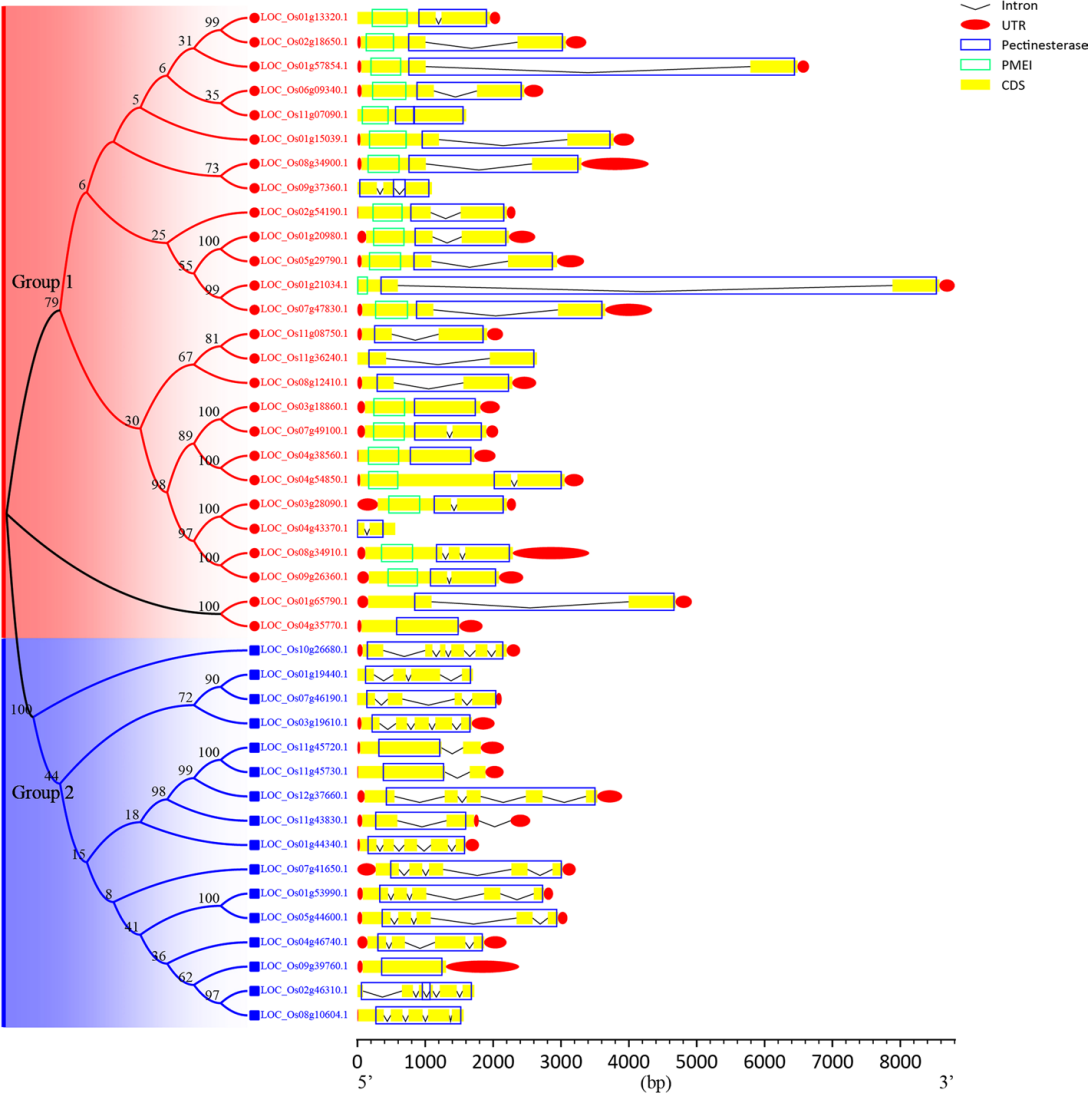
Phylogenetic tree, gene structure, and domain diagram of rice PMEs

The gene structure of rice PME is shown in Fig. 2 and the number of structural elements is shown in Table 1. We observed that the average length of rice PME gene is 2802.62 bp, the longest is 8802 bp (*LOC_Os01g21034.1*), and the shortest is 557 bp (*LOC_Os04g43370.1*); the average numbers of introns, cds, and utrs are 1.79, 2.76, and 1.69, respectively; the maximum values are 5 (*LOC_Os10g26680.1* and *LOC_Os02g46310.1*), 6 (*LOC_Os10g26680.1* and *LOC_Os02g46310.1*), and 3 (*LOC_Os11g43830.1*), respectively; and the minimum values are 0 (*LOC_Os11g07090.1*, *LOC_Os03g18860.1*, *LOC_Os04g38560.1*, *LOC_Os04g35770.1*, and *LOC_Os09g39760.1*), 1 (*LOC_Os11g07090.1*, *LOC_Os03g18860.1*, *LOC_Os04g38560.1*, *LOC_Os04g35770.1*, and *LOC_Os09g39760.1*), and 0 (*LOC_Os11g07090.1*, *LOC_Os09g37360.1*, *LOC_Os11g36240.1*, *LOC_Os04g43370.1*, *LOC_Os01g19440.1*, and *LOC_Os02g46310.1*), respectively.

**Table 1:** The basic statistical details on structural elements of rice PME genes

Group	Gene	Length of gene	Number of intron	Number of exon		Type of gene	Category
				CDS	UTR		
Group 1	*LOC_Os01g13320.1*	2103	1	2	1	Intron-poor gene	Intron-poor clade
	*LOC_Os02g18650.1*	3372	1	2	2	Intron-poor gene	
	*LOC_Os01g57854.1*	6655	1	2	2	Intron-poor gene	
	*LOC_Os06g09340.1*	2739	1	2	2	Intron-poor gene	
	*LOC_Os11g07090.1*	1602	0	1	0	Intronless gene	
	*LOC_Os01g15039.1*	4076	1	2	2	Intron-poor gene	
	*LOC_Os08g34900.1*	4290	1	2	2	Intron-poor gene	
	*LOC_Os09g37360.1*	1098	2	3	0	Intron-poor gene	
	*LOC_Os02g54190.1*	2328	1	2	2	Intron-poor gene	
	*LOC_Os01g20980.1*	2620	1	2	2	Intron-poor gene	
	*LOC_Os05g29790.1*	3339	1	2	2	Intron-poor gene	
	*LOC_Os01g21034.1*	8802	1	2	2	Intron-poor gene	
	*LOC_Os07g47830.1*	4342	1	2	2	Intron-poor gene	
	*LOC_Os11g08750.1*	2145	1	2	2	Intron-poor gene	
	*LOC_Os11g36240.1*	2646	1	2	0	intron-poor gene	
	*LOC_Os08g12410.1*	2634	1	2	2	Intron-poor gene	
	*LOC_Os03g18860.1*	2097	0	1	2	Intronless gene	
	*LOC_Os07g49100.1*	2074	1	2	2	Intron-poor gene	
	*LOC_Os04g38560.1*	2035	0	1	2	Intronless gene	
	*LOC_Os04g54850.1*	3333	1	2	2	Intron-poor gene	
	*LOC_Os03g28090.1*	2335	1	2	2	Intron-poor gene	
	*LOC_Os04g43370.1*	557	1	2	0	Intron-poor gene	
	*LOC_Os08g34910.1*	3415	2	3	2	Intron-poor gene	
	*LOC_Os09g26360.1*	2443	1	2	2	Intron-poor gene	
	*LOC_Os01g65790.1*	4928	1	2	2	Intron-poor gene	
	*LOC_Os04g35770.1*	1844	0	1	2	Intronless gene	
Group 2	*LOC_Os10g26680.1*	2398	5	6	2	Intron-rich gene	Intron-rich clade
	*LOC_Os01g19440.1*	1702	3	4	0	Intron-poor gene	
	*LOC_Os07g46190.1*	2126	3	4	2	Intron-poor gene	
	*LOC_Os03g19610.1*	2021	4	5	2	Intron-rich gene	
	*LOC_Os11g45720.1*	2159	1	2	2	Intron-poor gene	
	*LOC_Os11g45730.1*	2154	1	2	2	Intron-poor gene	
	*LOC_Os12g37660.1*	3901	4	5	2	Intron-rich gene	
	*LOC_Os11g43830.1*	2546	2	2	3	Intron-poor gene	
	*LOC_Os01g44340.1*	1791	4	5	2	Intron-rich gene	
	*LOC_Os07g41650.1*	3216	4	5	2	Intron-rich gene	
	*LOC_Os01g53990.1*	2883	4	5	2	Intron-rich gene	
	*LOC_Os05g44600.1*	3095	4	5	2	Intron-rich gene	
	*LOC_Os04g46740.1*	2197	3	4	2	Intron-poor gene	
	*LOC_Os09g39760.1*	2382	0	1	2	Intronless gene	
	*LOC_Os02g46310.1*	1722	5	6	0	Intron-rich gene	
	*LOC_Os08g10604.1*	1565	4	5	1	Intron-rich gene	
	Mean	2802.62	1.79	2.76	1.69		
	Max	8802	5	6	3		
	Min	557	0	1	0		

According to the number of introns, eukaryotic genes can be divided into three categories: intronless (no introns), intron-poor (three or fewer introns per gene), and intron-rich (more than three introns per gene) [10]. Combined with the phylogenetic relationship, we found that the genes in Group 1 are only intronless (4, 15.38%) and intron-poor (22, 84.62%). Therefore, Group 1 is intron-poor clade. The genes in Group 2 contain these three types of genes, among them, intron-rich is the most (9, 56.25%), followed by intron-poor (6, 37.50%), and the least is intronless (1, 6.25%). Therefore, Group 2 is an intron-rich clade.

Combined with the location of the domains, we found that introns are almost always present in the region encoding the pectinesterase domain, whereas introns are absent in the region encoding the PMEI domain. Intriguingly, for the region encoding the pectinesterase domain, the genes of Group 2 contain more introns, while the genes of Group 1 contain fewer introns.

In conclusion, CFVisual showed the structure of rice PME gene well and provided useful quantitative information, which promoted our understanding and evolution of rice PME gene structure.

**Fig. 3:**
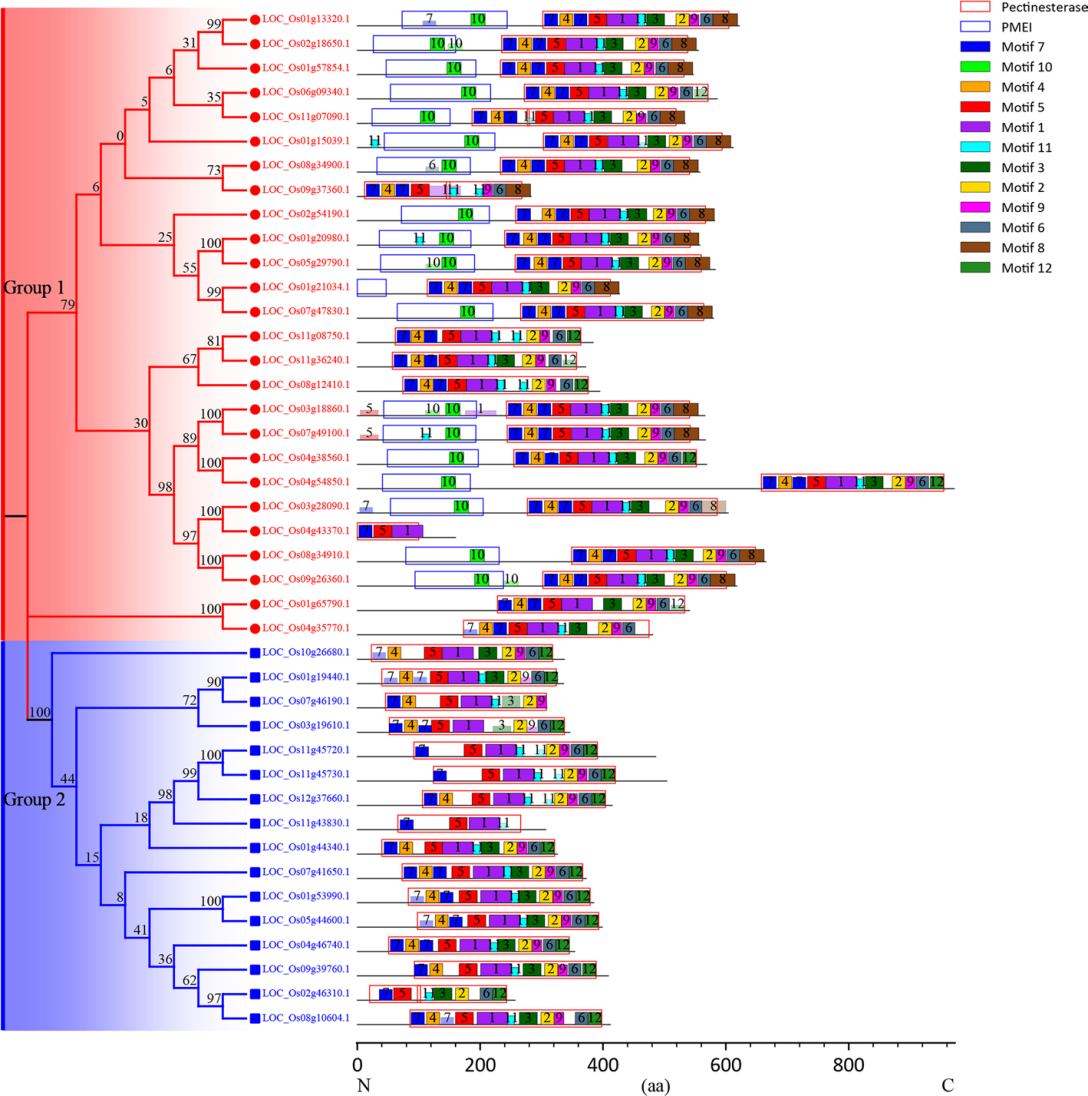
Phylogenetic tree, motif, and domain diagram of rice PMEs

The structural motifs and domains along a line representing the amino acid sequence were shown in Fig. 3. We found that motif 10 exists only in the PMEI domain, and is a sequence signature of the PMEI domain. Motif 7, motif 4, motif 5, motif 1, motif 11, motif 3, motif 2, motif 9, motif 6, and motif 12 are contained in the pectinesterase domain. Moreover, we also found some cases of motif repetition and loss, for example, motif 7 located in the pectinesterase domain has a repetition after motif 4, and the PME in Group 1 is relatively intact, while the PME in Group 2 is mostly missing. Interestingly, motif 8 and motif 10 are only present in PMEs in Group 1 and cannot be found in PMEs in Group 2. All in all, rice PME protein sequences are generally conserved and have some obvious differences. From a phylogenetic point of view, the distribution of motifs and domains has obvious specificity. This helps us to better understand the sequence characteristics and evolution of rice PME.

## Discussion

CFVisual can draw phylogenetic tree, gene structure, promoter *cis*-acting element, motif, and domain diagram, and stitch them in any form. The generated pictures can be directly used in the paper for display, allowing researchers to bid farewell to the retouching. CFVisual has been used by some researchers and cited by some articles [11–13]. In the future, it will become the best choice for researchers to draw gene structure and protein architecture.

## Data Availability

All data generated or analyzed during this study were included in this published article and the Additional files. We have been using public data and do not have produced sequence data by ourselves.
